# 

**Published:** 1888-02-18

**Authors:** 


### The Students' Column.

### UNIVERSITY OF LONDON.

### PRELIMINARY SCIENTIFIC (M.B.) EXAMINATION: JANUARY, 1888.

Examiners.—Chemistry: Prof. J. Emerson Reynolds, M.D, F.R.S., and Prof. W. A Tilden, D.Sc., F.R.S. Experimental Physics: R. T. Glazebrook, Esq., M.A., F.R.S., and Prof. A. W. Reinold, M.A, F.R.S. Biology: Prof. Bayley Balfour, M.D., D.Sc., F.R.S., Prof. F. O. Bower, D.Sc., M.A., Prof. E. Ray Lankester, M.A., F.R.S., and Adam Sedgwick, Esq., M.A., F.R.S.

PASS LIST.

Entire Examination.

First Division.—Griffiths, John Howell, University College, Aberystwith, and St. Bartholomew's Hospital; Soden, Wilfred Newell, St. Bartholomew's Hospital; Wesley, Frank William, University College.

Second Division.—Armitage, Fanny, Yorkshire College; Hall, James, B.A., Private study and University Corresp. College; Johnson, William John, Guy's Hospital; Jones, Arthur Bassett, Epsom and University Colleges; Mott, Clarence Harry, University College and University Corresp. College.

Two Subjects of the Examination ¶

Batteson, V. Jenner (C., B.), University College and Private study; Butcher, Charles (C., P.),† Guy's Hospital; Collyer, Brice (C., P.),† St. Bartholomew's Hospital; Eves, P. Stanhope (C., P.), University College and School; Jacob, Henry Wm. (C., P.), Trinity College, Dublin; Moore, Hugh M. (P., B.), Private study; Revell, Hugh Stanley (C., P.), University College and School; Rogers, Leonard (C., P.), St. Mary's Hospital; Turner, William (C., P.), King's College and School; Wells, John Edmund B. (C., B.), St. Mary's Hospital and Private tuition.

One Subject of the Examination ¶

Arnold, Gilbert James (C.), St. Thomas's Hospital; Bathurst, Lullm. Wood (P.),† St. Bartholomew's Hospital and Private study; Budgett, Alice Mc W. (C.),† University College; Carter, Frederick Jn. (P.),† St. Bartholomew's Hospital; Carwardine, Thomas (P.),† University College and Middlesex Hospital; Crawshaw, James Hy. (P.),† Archbishop Holgate's School, York, and Private tuition; De Korté, Wm. Edmond (B.),† University College; Jäger, Harold Julius (C.),† King's College; Lane, Harold William (C.), University College; Pace, Harold Everett (P.),† University College and London Hospital; Pantin, Charles Satchell (P.), Blackheath Proprietary School and University College; Peake, Arthur Walton (B.),† University College, Bristol; Piercy, Ann Frances (P.),† Queenswood School, Birkbeck Institution, and Private study; Rowland, Frederick Rowland (B.),† University Collge; Stokes, Whitley (P.), Trinity Hall, Cambridge, and King's College; Taylor, Herbert Stockley (C.), University College; Tildesley, J. Percival (P.),† Mason College, Birmingham; Vickers, K B. James (C.), Westminster School and St. Thomas's Hospital.

¶ The subjects taken by these Candidates are indicated by initials after the name—C.=Chemistry; P.=Physics; B.=Biology.

† These Candidates have now completed the Examination.

### INTERMEDIATE EXAMINATION IN MEDICINE: JANUARY, 1888.

Examiners.—Anatomy: Prof. D J. Cunningham, M.D., C.M., F.R.S.E., and Prof. Curnow, M.D. Physiology: Prof. E. A. Schäfer, F.R.S., and Prof. Gerald F. Yeo, M.D. Materia Medica, &c.: J. Mitchell Bruce, Esq., M.D, M.A., and T. Lauder Brunton, Esq., M.D., D.Sc., F.R.S. Organic Chemistry: Prof. J. Emerson Reynolds, M.D., F.R.S., and Prof. W. A. Tilden, D.Sc., F.R.S.

PASS LIST.

Entire Examination.

First Division.—Ballance, Herbert Stanley, King's College; Berry, Albert Edward Owens College; Boning, Arthur Wilson, University College; Box, Charles Richard, St. Thomas's; Dodwell, Philip Rashleigh, University College; Gill, James McDonald, Guy's; Hall, Frederick William, Guy's; Hugo, Edward Victor, St. Bartholomew's; Lord, Robert Ellis, B.Sc., Owens College; Lyons, Algernon Wilson, King's College; Playfair, Hugh James Moore, King's College; Sandifer, Henry Stephen, King's College; Sykes, John Herbert, Owens College.

Second Division.—Cass, Arthur Morgan, Owens College and Manchester Royal Infirmary; Dawson, Bertrand Edward, London Hospital and University College; Distin, Howard, King's College; Dove, Percy William, St. Bartholomew's; Earle, Edward Robert Charles, University College; Girling, Charles John, Guy's; Johnston, George Saint, Queen's College, Birmingham; Kitchin, Henry Brunton, University College; Ricketts, Thomas Frank, B. Sc., Guy's; Roberts, Llewelyn, St. Bartholomew's.

Excluding Physiology,

First Division.—Clark, William Adams, St. Bartholomew's.

Second Division.—Pridmore, Eric Leonard Norman, University College; Waring, John Alfred, University College.

Physiology only.

First Division.—Lea, Arnold William Warrington, Owens College; Morgan, Samuel Wolferstan, Bristol Medical School; Pickels, Joseph Arthur, Owens College.

Second Division.—Hall, Elias George, Bristol Medical School; Langdale, Henry, Owens College; Taylor, Frederic Ryott Percival, Westminster.

### SPECIAL NOTICE TO NEWSAGENTS, ADVERTISERS, AND THE TRADE.

### CHANGE OF OFFICE AND PUBLISHER.

NOTICE is hereby given, that the Office of THE HOSPITAL will henceforth be at 38, Old Jewry (Cheapside end), E.C., to which address all communications should be sent. Mr. A. Doig has been appointed Advertising Agent, and Mr. J. H. S. Hanning, Manager. All Cheques and Post Office Orders should be made payable to J. H. S. Hanning, 38, Old Jewry, E.C., crossed Lloyds, Barnetts, and Bosanquets Bank (Limited).

### NURSING MIRROR.

Several communications for the "Mirror" and "Editor's Letter Box" are unavoidably held over until next week in consequence of the great pressure on our space.

### Amusements with Profit for all Ages.

**Rules.**—All answers must be addressed to the Prize Editor, The Hospital, 38, Old Jewry, Cheapside, London, E.C., not later than the *first* post on Thursday next. The full name and address must in all cases be given, but a *nom de plume* may be added for publication. Only one side of the paper must be written on.

### FOR MEN AND WOMEN.

### Acrostic Competition.

Commenced November 26th, 1887; ends February 18th, 1888.

A PRIZE OF ONE GUINEA will be given to the competitor who gains the highest number of marks for best original acrostics during the ensuing quarter. All acrostics sent in for this competition will be credited according to merit, twelve marks being the highest score given for one acrostic.

A PRIZE of HALF-A-GUINEA to be given to the competitor who gains the highest score for solution of acrostics set, including the one inserted on November 26th. One mark only will be given to the competitors who solve all the lights of each acrostic.

These competitions to alternate weekly.

Credit will be given this week for solution.

No. 7. Double Acrostic.

Such bliss they deem my *first*, who aye secure
In Castle Indolence drone life away,
Whom learning's ample page doth ne'er allure.
Nor wonder, child of nimble fancy's play,
Enticeth to the quest of Nature's sway,
That even in their brutish soul they trow
My *finals* to be folly, foolish they,
As though a beast's existence were enow
For men, whom Allwise God doth with a mind endow.

Dipt in this well, the barbs of hate
Wound hearts, men's peace destroy;
Drops from this well communicate
Hearts ease by spells of joy.

A remarkably eminent man,
Who rode a remarkable race,
Which he irresponsibly ran
At a quite reprehensible pace.

Old Fuller calls him reverently

"God's Image cut in ebony."

"Under the greenwood tree"
My life is merry and free.
My lord's fat buck and the Churchman's pelf
Are mine by no favour; I help myself.

Queen of the Parisian stage,
The wonder, idol of her age.
The Old and New World own'd her hour
Of mimic Passion's moving power.

He was no spy! the true and brave
Albeit he found a felon's grave.

Thus "weariness can snore upon the flint,"
Or in the straight-backed chair.
So Jove was fabled, by this sovereign hint,
To shake earth, sea, or air.

As ivy to the elm,
So the cliff climber to his rope;
As the brave steersman to his helm,
So hearts to hope.

"Fair was she to behold, that maiden of seventeen [summers."

### Answers.

No. 6. Double Acrostic.

Evening—Morning.

| | | | |
|---|---|---|---|
| E | lysiu | M | Mrs. Hemans. |
| V | elin | O | Byron, "Childe Harold, Canto IV." |
| E | leano | R | Tennyson, "Dream of Fair Women." |
| N | elso | N | Scott, "Introduction to Marmion." |
| I | Zingar | I | Bolland, "Cricketing Song." |
| N | apoleo | N | Byron, "Lines from the French." |
| G | loamin | G | Milton, "Paradise Lost." |

Correct answers have been received from Dunce, Condorcet, Pasteur.

Second Quarterly Competition commenced February 4th, 1888; ends April 28th, 1888.

Three prizes of one guinea, 15*s.* and 10*s.* 6*d.* will be given each quarter to the three competitors who obtain the highest number of marks: (1) by making the largest number of words derived from the word or phrase set for anatomical dissection; or (2) for the best answers to (B); or (3) by (1) and (2) combined.

### (A) THE WORD COMPETITION.

We shall give the newspapers and periodicals for dissection during this quarter, that for this, the *third* week of the quarter, being

"The World"

Observe.—Proper names, abbreviations, foreign words, words of less than four letters and repetitions are barred. Nuttall's *Standard* dictionary only to be used.

N.B.—All words sent in must be arranged *alphabetically* with correct total affixed.

### Results of First Week.

*Advertiser.*—Accepted scores: Patience (397), Gretta (356), Ellice (351), E. E. (273), Ninny (259), Rowdy Corner 254), Isabel (238), Southwark Owl (232), Condorcet (230), Lodge (222), Pigeon (216), Brisbane (215), Qui vive (213), Susan Nipper (212), Dunce (207), Lightowlers (187), Lauriston (185), Sym (184), Lancashire Witch (181), Nelle (179), K. M. (174), H. L. J. (136), Vivo (126).

### (B) THE LITERARY COMPETITION.

Some people hate puzzles of all kinds, including word competitions, but they are fond of correspondence, and many sisters and mothers are continually receiving and sending letters to and from members of the family who are abroad in India or the Colonies. The constant coming and going between England and other portions of the British Empire make it a matter of interest and importance to know something about the life, the climate, the amusements, the adventures, the clothing, and the surroundings of those who seek a livelihood beyond the seas. We shall therefore try to find space for the publication of selected letters from abroad, which may be sent by any of our readers who have friends and relations in foreign lands. We shall give marks for these letters as well as for the word competitions which will count equally for these prizes, so that anyone may enter for both or either, the prizes to be given to those who get the highest number of marks.

*Gretta* and *L'Americaine* send letters this week.

### Letters from Across the Seas.

L'Americaine writes from Seville.

Here we are in the South of Spain, a cloudless blue sky above us, and the air warm and balmy. We entered Spain from the South of France, starting from Biarritz. Our first halt in Spain was at Burgos. We visited the lovely old cathedral, and saw, amongst other things, the bones of the Cid, who was born there. The hotels are wretched, and we felt that having once seen the town, we should have no wish to return to it. From Burgos we went to Valladolid; here again the churches are in the most exquisite style of gothic architecture. The cathedral is very old. A festival was being held in honour of Santa Teresa, a saint much honoured throughout Spain. Whenever there is a saints day all the shops are closed, and the towns look so dreary. From Valladolid we went to Madrid, which is not a bit Spanish in outward appearance, it is like a pocket edition of Paris, a very lively and bright capital, with the most hideous dried-up country all round it. We enjoyed the pictures in the museum greatly. The Murillos are exquisite, and the collection of paintings is considered one of the finest in the world. I know that we all enjoyed it far more than the galleries of Florence or Dresden. Mr. B— has just returned from a bull fight. I will not give you the details, but he says it is the most brutal and repulsive thing imaginable. Next week there is to an unusually grand one, and Queen Isabella is expected to be present. Of course we went to see the Escurial, a combination of palace, convent and church, built by Philip, Queen Mary's husband. It is a big plain building, but there is a certain grandeur about its simplicity; here the Spanish sovereigns are buried—a gloomy place to rest in. From Madrid we made an excursion to the interesting old town of Toledo, which abounds in Moorish remains. The mosques are now turned into churches, alcazars (palaces) are used as public offices and dwelling houses. Our next move was to Seville; after travelling all night we found ourselves in a different climate and among very different scenes. This is a curiously built town with narrow winding streets—each house has a patio or inner court, where oranges or bananas, etc., grow. In the summer, which is intensely hot, the people bring their mattresses out into the courts, which are much cooler than the upper rooms of the houses, and sleep and live there. These courts are beautifully paved with coloured tiles, and generally there is a fountain playing in the middle which keeps the air delightfully fresh and cool. On the 2nd we went to the cemetery to see the people decorate the graves of the dead, which they do every year on this day. It was a curious sight—some were kneeling before a grave praying and weeping, others were arranging flowers on the tombs, whilst others again were walking about making it a high day and holiday. The Spaniards bury their dead in little niches made of brick or stone. The women of Seville are very handsome. Their usual dress is black with the lace mantilla, arranged with perfect grace and coquetry. It is hopeless for a foreigner to don it with the idea of passing off for a native, for they can tell at a glance by the way the mantilla is arranged, and her mode of using the indispensable fan that she is not a Spaniard.

### THE CHILDREN'S CORNER.

PRIZES.

FOR MOST CORRECT ANSWERS TO PUZZLES.

This Commenced October 1st, 1887.

One Pound a Month.

A PRIZE OF THREE GUINEAS

to the Competitor who shall have sent in the largest number of correct or best answers during the twelve months.

### Puzzles—Third Week.

No. 9. Enigma.

Blind we are, blind from our birth,
Yet we have eyes tho' we live in the earth,
Eyes that were never intended to see
Can hardly if any great use to us be.

No. 10. Geographical Diamond Puzzle.—1. A consonant. 2. A Swiss river. 3. Islands to the North of Scotland. 4. A well known English fishing port. 5. A town in Germany. 6. An island off Hampshire. 7. A country in Europe. 8. Mountain range in Asia Minor. 9. A consonant.

No. 11. Make a sentence out of the following:—

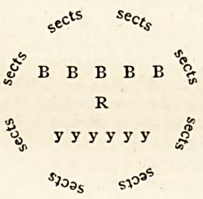

No. 12. Query.

Though letters five compose my name,
Remove the *first*, I'm still the same,
But take the *first* and *next* away,
Then only *one* behind will stay.

### Results of First Week Puzzles.

No. 1. Historical Charade.—Can-more=Canmore.

No. 2. Dessert Enigmatically Expressed.—1. Ap-ri-cot=Apricot. 2. Oranges. 3. Prune, or pare =(pear). 4. Pine-apple=Pineapple.

No. 3 Transposition.

Stone—Tone—Note—Eton.

No. 4. Calico.

*All right*—Gem, Alsie. A. C. K., Alert, M. S.W., Poodle, Excelsior, A. G. S., McCulloch, Spider, Druce, Jerusalem, Happy Eliza, Judy, Oswald H., Moina, Squeaker, Plummy Fluff, Scrooge, Robinson. *Three right*—Toby, Mount Edgecumbe. *Two right*—Betty Burke.

All competitors who send in papers must strictly abide by the following

### Conditions.

I. Every paper sent in must be clearly written, and one side only must be used. All competitors must mark at the head of their papers the number of their competition.

II. Each paper must be signed by the author with his or her real name and address. A *nom de plume* may be added, if the writer does not desire to be referred to by us by his real name. In the case of all prize-winners, however, the real name and address will be published.

III. All communications relating to prize competitions should be addressed to "The Prize Editor, The Hospital, 38, Old Jewry, London, E.C." Competitors are requested not to include in letters, etc., to the Prize Editor communications on matters of other business. All letters must be fully prepaid.

IV. The Prize Editor of "The Hospital" is the sole judge of the competitions, and his decision is in all cases final and without appeal.

V. The Prize Editor reserves the right to publish any paper sent in, whether it receives a prize or not. He does not hold himself responsible for any MSS. sent, nor can he undertake to return rejected competitions.

**Notice to all Correspondents.**—N.B.—All letters referring to this page, which do not arrive at 38, Old Jewry, Cheapside, London, E.C., by the *first post on Thursdays*, and are not addressed PRIZE EDITOR, will in future be disqualified and disregarded.

No one will be permitted to win the Monthly or Quarterly Prizes twice in any one year, the annual prizes being offered especially to prevent this.

